# The *Drosophila* orthologue of progeroid human WRN exonuclease, DmWRNexo, cleaves replication substrates but is inhibited by uracil or abasic sites: Analysis of DmWRNexo activity in vitro

**DOI:** 10.1007/s11357-012-9411-0

**Published:** 2012-05-05

**Authors:** Penelope A. Mason, Ivan Boubriak, Timothy Robbins, Ralph Lasala, Robert Saunders, Lynne S. Cox

**Affiliations:** 1 Department of Biochemistry, University of Oxford, South Parks Road, Oxford, OX1 3QU UK; 2 Department of Life Sciences, The Open University, Milton Keynes, MK7 6AA UK

**Keywords:** WRN, Werner syndrome, Exonuclease, Ageing, RecQ, DmWRNexo, Progeroid syndromes, DNA replication, DNA repair, DNA recombination

## Abstract

Werner syndrome (WS) is a rare late-onset premature ageing disease showing many of the phenotypes associated with normal ageing, and provides one of the best models for investigating cellular pathways that lead to normal ageing. WS is caused by mutation of *WRN*, which encodes a multifunctional DNA replication and repair helicase/exonuclease. To investigate the role of WRN protein’s unique exonuclease domain, we have recently identified DmWRNexo, the fly orthologue of the exonuclease domain of human WRN. Here, we fully characterise DmWRNexo exonuclease activity *in vitro*, confirming 3′–5′ polarity, demonstrating a requirement for Mg^2+^, inhibition by ATP, and an ability to degrade both single-stranded DNA and duplex DNA substrates with 3′ or 5′ overhangs, or bubble structures, but with no activity on blunt ended DNA duplexes. We report a novel active site mutation that ablates enzyme activity. Lesional substrates containing uracil are partially cleaved by DmWRNexo, but the enzyme pauses on such substrates and is inhibited by abasic sites. These strong biochemical similarities to human WRN suggest that *Drosophila* can provide a valuable experimental system for analysing the importance of WRN exonuclease in cell and organismal ageing.

**Electronic supplementary material:**

The online version of this article (doi:10.1007/s11357-012-9411-0) contains supplementary material, which is available to authorized users.

## Introduction

Werner syndrome, a rare but highly informative premature ageing syndrome, is caused by mutation of the human *WRN* gene (Yu et al. 1996) which encodes a large protein (hWRN) possessing both helicase and exonuclease activities (Gray et al. 1997; Huang et al. 1998; Shen et al. 1998). WS patients show premature onset of many signs of normal human ageing including athero- and arterio-sclerosis and type II diabetes together with high cancer incidence (Cox 2008; Epstein et al. 1966; Goto 2001). Genetically, WS patient cells show karyotypic abnormalities with DNA rearrangements including translocations and deletions (Fukuchi et al. 1989; Scappaticci et al. 1982).

The human WRN protein is involved in many aspects of DNA metabolism including DNA repair (Bohr 2005), DNA replication (Pichierri et al. 2001; Rodriguez-Lopez et al. 2002; Sidorova et al. 2008) and DNA recombination (Saintigny et al. 2002, reviewed in Cox and Faragher 2007; Kudlow et al. 2007). The exonuclease activity of hWRN has been implicated in DNA repair using deletion mutants (Kashino et al. 2005), while single point mutations in either the exonuclease or helicase domain (or both) suggest separable but critical roles in recombination and cell survival (Swanson et al. 2004). The high incidence of stalled replication forks in WS cells (Rodriguez-Lopez et al. 2002; Sidorova et al. 2008), together with hypersensitivity of WS cells to 4-nitroquinoline oxide and camptothecin (Christmann et al. 2008; Lebel and Leder 1998; Ogburn et al. 1997; Pichierri et al. 2000; Poot et al. 1999; Prince et al. 1999; Rodriguez-Lopez et al. 2007), agents that result in stalled or collapsed replication forks (respectively), suggest that WRN is required either to prevent formation of hyper-recombinant replication intermediates when DNA replication is interrupted, or to resolve such structures when they form. Moreover, the S phase defects and CPT sensitivity of human WS cells can be overcome by ectopic expression of a Holliday junction nuclease (Rodriguez-Lopez et al. 2007). Taken together, these findings suggest that the WRN exonuclease plays an important role in maintaining genome stability through several DNA metabolic pathways.

In vertebrate WRN, one polypeptide contains both the exonuclease and helicase activities; in other organisms, the two functions are encoded by separate genetic loci (Plchova et al. 2003). We have recently identified and cloned the WRN exonuclease orthologue in the fruit fly *Drosophila melanogaster*, DmWRNexo (encoded by the *Drosophila* gene *CG7670*, Cox et al. 2007), and demonstrated genetic instability in hypomorphic *CG7670* mutants (Saunders et al. 2008). For direct analysis of the exonuclease distinct from helicase activity, we analysed the activity of purified recombinant DmWRNexo, which entirely lacks helicase domains, and showed that the protein does indeed function as an exonuclease (Boubriak et al. 2009). Here, we provide a thorough analysis of the enzymatic activities of DmWRNexo: we assess concentration dependence and processivity of DNA cleavage by DmWRNexo, its buffer and divalent cation specificities, and its cleavage activity on substrates including DNA bubbles and duplexes with recessed 5′ or 3′ ends, together with substrates containing either uracil or an abasic site. Our results demonstrate that the wild-type enzyme has low processivity, with an unequivocal 3′–5′ polarity, and a requirement for Mg^2+^. We show that a novel active site mutation (D222V) ablates nuclease activity and investigate how a mutation that alters the surface fold of the protein (D229V) severely abrogates exonuclease activity on a range of substrates. We further show that wild-type DmWRNexo can cleave substrates resembling replication intermediates, including DNA bubbles and duplex overhangs, but that the enzyme pauses on damaged substrates at uracil and is unable to cleave beyond abasic sites. The distinct similarities between the exonuclease activities of hWRN and DmWRNexo that we report here extend the use of *Drosophila* as a powerful system enabling cellular and organismal analysis of the role of WRN in DNA metabolism, development and ageing.

## Materials and methods

### DNA substrate preparation

DNA substrates (Table 1, Fig. S1) were annealed at a 3:2 ratio of unlabelled guide strand/labelled oligonucleotide in 1× TE/50 mM NaCl (95°C for 3 min, cooled to rt) to a final concentration of 250 μM (labelled oligonucleotide) and verified by PAGE analysis (Fig. S1). To make abasic (AP) sites, oligonucleotides containing a single uracil residue were treated with uracil DNA glycosylase and substrates prepared as above. AP sites were confirmed by conversion to breaks (Higurashi et al. 2003) (Fig. S1).

**Table 1:**
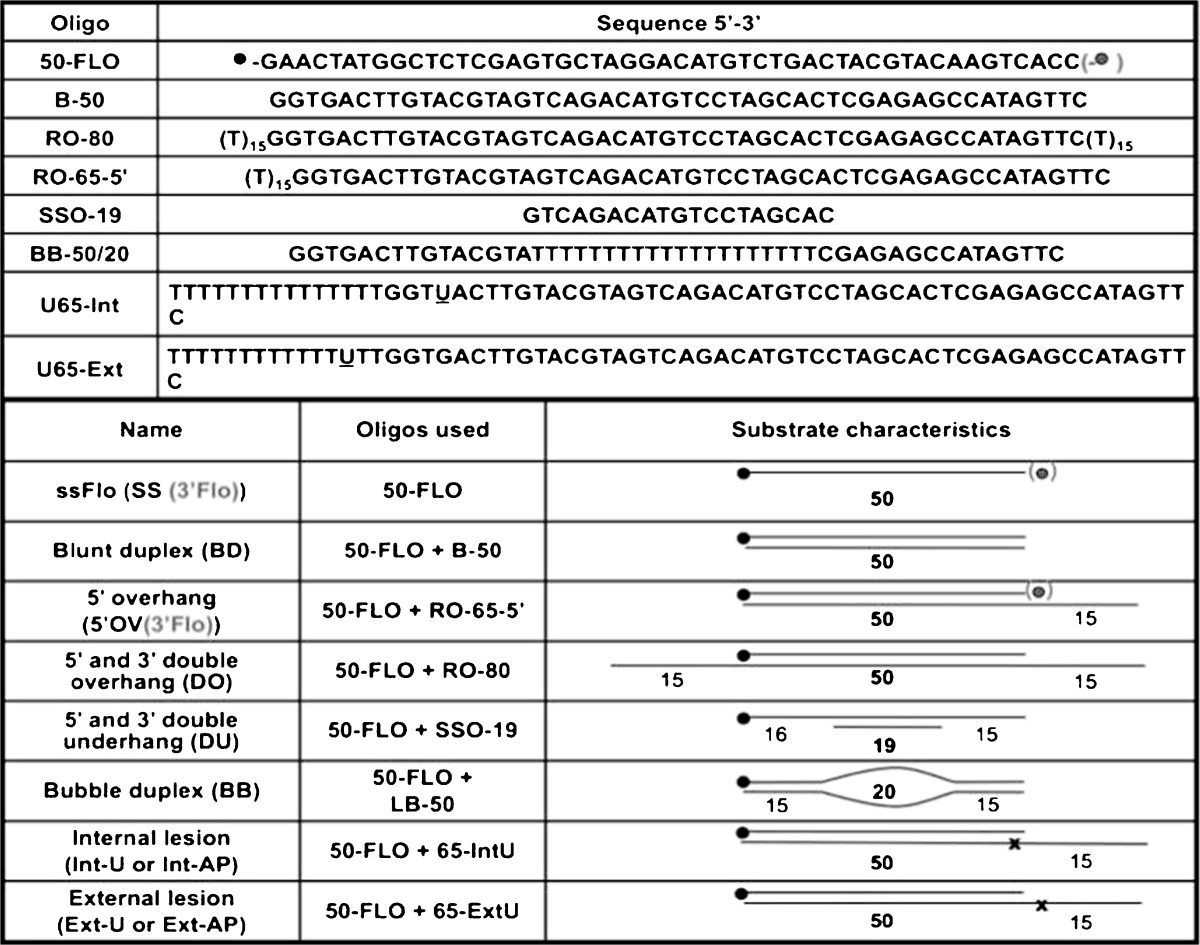
Oligonucleotides used in this study

### Bioinformatics and molecular modelling

Protein sequence alignments (DmWRNexo/hWRN exonuclease domain) utilised BLAST (Altschul et al. 1990, 1997). A putative active site residue was identified at aspartate 222, and a D222V mutation was created by site-directed mutagenesis of *CG7670* cDNA using the primers A665T 5′-CGTGAACATAAAGAACGTTTTCCGAAAGCTGGCAC-3′ and A665T-antisense 5′-GTGCCAGCTTTCGGAAAACGTTCTTTATGTTCACG-3′ according to manufacturer’s instructions (Quickchange, Stratagene). Predicted structures of DmWRNexo variants (WT; D162A, E164A; D222V and D229V) were modelled against hWRN (Perry et al. 2006) using SWISS-MODEL (Arnold et al. 2006; Kiefer et al. 2009; Peitsch et al. 1995) and MacPyMol v 0.99 (Delano Scientific).

### Recombinant protein expression and purification

Mock, WT, mutant D229V, double mutant (D162A, E164A) (Boubriak et al. 2009) and the new D222V DmWRNexo proteins were expressed, purified and analysed by SDS–PAGE and Western blot as described previously (Boubriak et al. 2009). Proteins were stored at −80°C with the addition of 20% glycerol in the storage buffer.

### Exonuclease assays

Exonuclease assays were conducted as described previously (Boubriak et al. 2009). Briefly, purified proteins (12.5–200 nM) were incubated with 2 μM DNA substrate in WRN exo buffer (hereafter called ‘Exo buffer’—40 mM Tris–HCl, pH 8.0, 4 mM MgCl_2_, 5 mM dithiothreitol, 0.1 mg/ml BSA) at 37°C for 30 min (Opresko et al. 2001) unless otherwise stated, and reactions stopped using 1:1 vol formamide buffer (80% formamide, 0.5× TBE; Opresko et al. 2001). Products were resolved and quantified as described (Boubriak et al. 2009). Competition analysis utilised increasing amounts of unlabelled substrate identical in sequence and structure to the labelled substrate, but lacking any FLO label.

### Cation and ATP assays

EDTA experiments contained Exo buffer with the addition of EDTA (0–8 mM). Cation substitutions (default 4 mM; chloride salt) in Exo buffer replaced MgCl_2_, as indicated in individual figures. Where relevant, ATP or analogues AMP-PNP or ATPγS were added to 2 mM. For ATP/magnesium competition, various concentrations of ATP and MgCl_2_ were tested in combination in a standard nuclease assay.

## Results

### DmWRNexo degrades both ss and ds DNA in a concentration-dependent manner

**Fig. 1:**
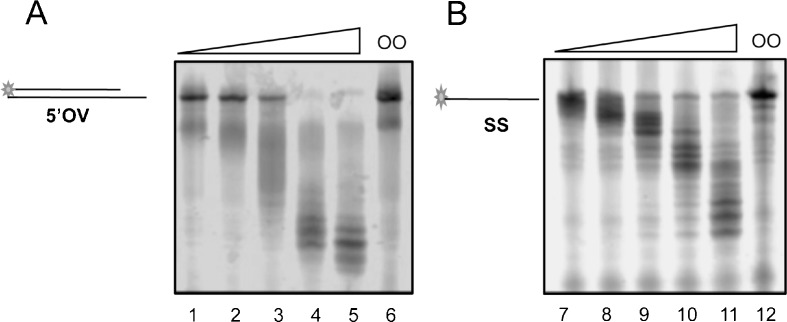
Concentration-dependent DNA cleavage by DmWRNexo. **a** Nuclease activity on a 5′-overhang (5′OV) double-stranded 5′-tailed substrate and **b** activity on a single-stranded DNA substrate (ss). Samples were separated on denaturing 14% PAGE. DmWRNexo protein was used at the following concentrations: *lanes 1*, *7* = 12.5 nM, *lanes 2, 8* = 25 nM, *lanes 3, 9* = 50 nM, *lanes 4*, 10 = 100 nM, *lanes 5*, *11* = 200 nM. OO (oligo only, *lanes 6*, *12*) have no protein added (see Fig. S2a for quantification)

To determine optimal molar ratios for analysis of DmWRNexo cleavage of single-stranded and duplex DNA substrates *in vitro* for subsequent experiments, we assessed the concentration dependence of DNA cleavage by purified recombinant DmWRNexo using a fluorescence-based assay we have developed (Boubriak et al. 2009). Increasing amounts of DmWRNexo protein (12.5–200 nM) were incubated with two different DNA substrates: duplex DNA with a 5′ overhang on the fluorescently labelled reporter strand (5′OV), or single-stranded (ss) DNA (see Table 1 and Fig. S1a for substrates used). DmWRNexo cleaved both ssFLO and 5′OV duplex DNA substrates in a concentration-dependent manner (Fig. 1a, b), with greatest cleavage achieved at 200 nM protein. The degradation profiles of both single-stranded and duplex substrates were comparable (ss—*R* ^2^ = 0.94, 5′OV—*R* ^2^ = 0.96, see Fig. S2a), though initial loss of full-length substrate was more rapid for the duplex than single-stranded DNA. Interestingly, DmWRNexo proficiently degraded large amounts of DNA; at molar ratios of 80 times less protein than DNA, some degree of nuclease activity was observed, though with lower processivity. Based on these results, DmWRNexo protein concentrations of between 50 and 200 nM were subsequently used.

### Relative activity and processivity of WT DmWRNexo and mutant D229V

**Fig. 2:**
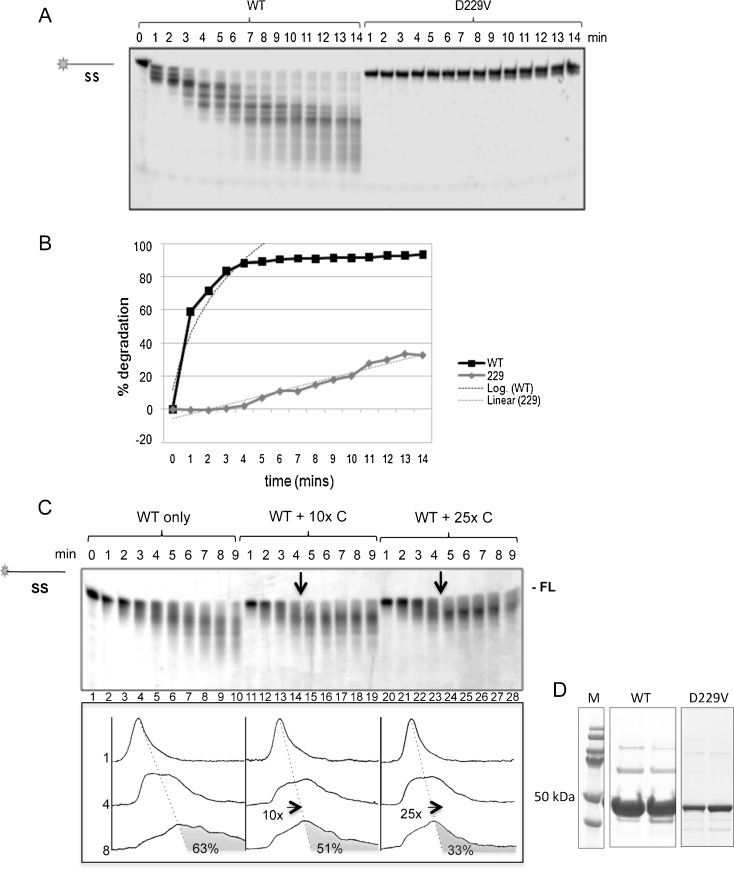
Rate of degradation and processivity of DmWRNexo. **a** Time course of degradation of single stranded DNA substrate (2 μM) incubated with 100 nM WT DmWRNexo or D229V mutant protein at 37°C over a 14-min time course in a total reaction volume of 80 μl; 5 μl of sample was removed into formamide dye every minute for analysis on denaturing PAGE. **b** Activity was quantified using ImageJ and plotted ±SEM (*n* = 3 for WT, *n* = 2 for D229V). Best-fit regression for each is shown (*dotted lines*—see text for *R* ^2^ values)—note linear regression for D229V compared with logarithmic curve for WT DmWRNexo. **c** Processivity on addition of unlabelled competitor substrate (*arrows*). *Upper panel*: denaturing PAGE of degradation products ± competitor DNA (*10× C* = 10-fold excess competitor DNA; *25× C* = 25× excess competitor DNA) compared with control without competitor (WT only). *Lower panel*: ImageJ quantification of degradation at 1, 4 and 8 min. *x* axis represents position migrated down the gel, *y* axis shows DNA fluorescence intensity (area under the curve). *Dotted lines* indicate relative gel position normalised to account for gel ‘smile’. *Shaded regions* represent degradation occurring after competitor was added, or equivalent time in ‘WT only’ control. **d** Coomassie blue staining of WT and D229V DmWRNexo following purification and separation on SDS–PAGE

We previously reported identification of a mutant of *CG7670* that alters a predicted surface residue aspartate 229 to valine. In flies, this mutation increased rates of recombination, suggestive that the enzyme was dysfunctional, which was verified using *in vitro* cleavage assays (Boubriak et al. 2009). Here, we have further investigated the D229V mutant, compared with wild-type DmWRNexo, by assessing cleavage of ssDNA substrate over a time course of 14 min. As shown in Fig. 2a, WT DmWRNexo efficiently degraded the substrate DNA whilst the D229V mutant showed a distinct single base clipping which never proceeded further. Quantification of degradation (Fig. 2b) allowed a crude estimate of the rate of activity for both proteins. Since the WT protein cleaves multiple times in each substrate, the cumulative degradation fitted a logarithmic curve [*f*(*x*) = 0.49 ln(*x*) + 0.11 (*R* ^2^ = 0.92)], whilst the single clipping activity of the D229V mutant fitted a linear regression [*f*(*x*) = 0.03*x* = 0.08 (*R* ^2^ = 0.96)]. This suggested that wild-type DmWRNexo has some, albeit limited, processivity, whilst the D229V mutant is totally non-processive.

Exonuclease processivity was further investigated using a competition assay with a non-labelled oligonucleotide otherwise identical to the ssFLO substrate (Table 1). The addition of 10-fold excess unlabelled competitor resulted in marked abrogation of cleavage, while addition of a 25-fold excess of competitor DNA immediately halted degradation of labelled template (Fig. 2c), suggesting that WT DmWRNexo readily dissociates from its substrate and hence has low processivity on a single-stranded template. It is possible that the enzyme must reposition itself on the substrate after each cleavage event.

Human WRN exonuclease is reported to have poor processivity that is enhanced by multimerisation (Perry et al. 2010). We therefore investigated the ability of purified recombinant DmWRNexo to form oligomers. In gel filtration analysis, DmWRNexo eluted in several peaks consistent with monomer, trimer and large aggregates (data not shown). Coomassie staining of the purified proteins on SDS–PAGE showed the rapid formation *in vitro* of multimers of WT DmWRNexo that were stable on heating and under reducing conditions; such higher molecular weight bands were not detected for D229V (Fig. 2d). Hence, low processivity of WT DmWRNexo is not a consequence of failure of oligomerisation *in vitro*, though it is conceivable that the inability of the D229V mutant to cleave beyond one nucleotide on a single-stranded substrate may be due to a problem in forming correct protein–protein interactions due to its surface alteration (see Fig. S3c).

### DmWRNexo recapitulates the 3′–5′ activity of human WRN

**Fig. 3:**
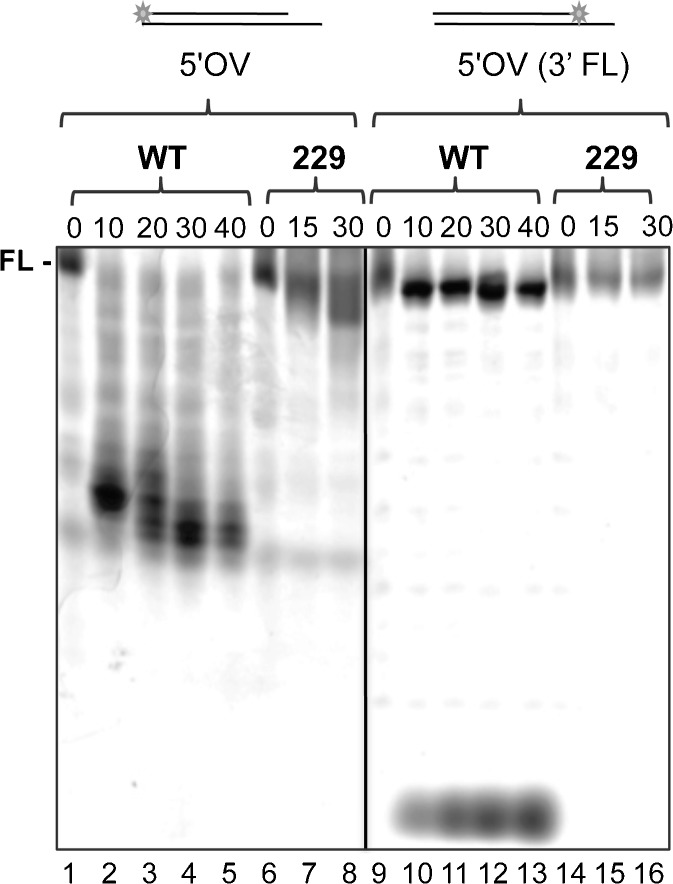
Exonuclease polarity of DmWRNexo. Time course of nuclease activity of WT DmWRNexo and the D229V mutant protein on 5′-labelled (5′ overhang substrate labelled with fluorescein at the 5′ end—5′OV) or 3′-labelled substrate [fluorescein on the recessed 3′ nucleotide (5′OV (3′FL)]. (See Fig. S2b for quantification)

Human WRN has 3′–5′ polarity as an exonuclease (Shen et al. 1998). Our preliminary studies with DmWRNexo suggested the same polarity, since a ladder of products was observed with 5′ labelling of a reporter strand (Boubriak et al. 2009). To unequivocally determine nuclease polarity, we compared cleavage of the standard 5′ overhang duplex substrate labelled on the 5′ end of the reporter strand (5′OV) with a 5′-tailed duplex substrate constructed with the fluorescein label conjugated to the 3′ recessed end [5′OV(3′FL), Fig. 3]. As expected, DmWRNexo degraded the 5′ end-labelled duplex substrate efficiently over 40 min, giving rise to a ladder of labelled products of decreasing size, indicating progressive cleavage from the 3′ end of the reporter strand (Fig. 3, lanes 1–5). Hence, activity consistent with a 3′–5′ exonuclease is observed.

A *bona fide* 3′–5′ exonuclease would be expected to clip off the fluorescein-labelled nucleotide of the 3′-labelled substrate, resulting in a single nucleotide product with high mobility in gel electrophoresis. Within 10 min of incubation of the 3′-labelled substrate with DmWRNexo nuclease, a band at the bottom of the gel representing a single nucleotide was observed, which increased in intensity with time, though no intermediate sized fragments were detected above background (Fig. 3, lanes 9–13). This 3′ single nucleotide clipping confirms that DmWRNexo is indeed a 3′–5′ exonuclease.

Quantification of full-length substrate remaining (Fig. S2b) shows that the majority was degraded when 5′ end-labelled, while only 40% of the 3′ end-labelled substrate was cleaved. This suggests that the fluorescein label may partially inhibit nuclease digestion by DmWRNexo, possibly because of steric hindrance (this is not observed for bacteriophage λ exonuclease, see Fig. S4). Most importantly, DmWRNexo exhibits 3′–5′ polarity with no evidence of any 5′–3′ activity.

These templates allowed further analysis of the D229V mutant protein. Over a 30-min time course, this mutant protein demonstrated limited degradation of the 5′-labelled duplex overhang substrate (Fig. 3, lanes 6–8), consistent with our previous observations, but unlike WT, the mutant enzyme showed no clipping of a 3′ end-labelled substrate (Fig. 3, lanes 14–16), suggesting that the fluorescein moiety *does* block the 229 mutant.

### DmWRNexo can use Mn^2+^ and Mg^2+^ as divalent cation

**Fig. 4:**
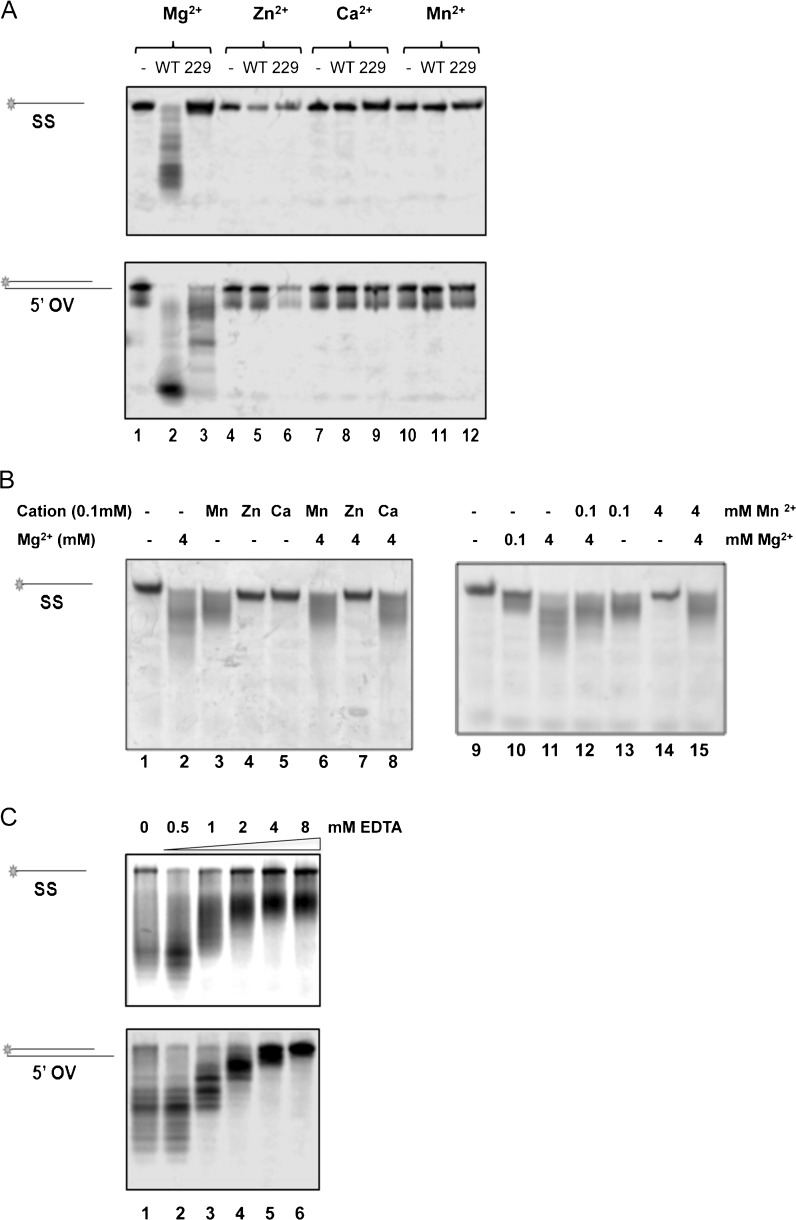
DmWRNexo preferentially requires magnesium for catalysis. **a** Nuclease activity on single-stranded substrate (SS, *upper panel*) or duplex overhang (5′OV, *lower panel*) of WT or mutant D229V (229) DmWRNexo protein (or no protein ‘−’, lanes 1, 4, 7 and 10) with 4 mM either Mg^2+^, Zn^2+^, Ca^2+^ or Mn^2+^. **b** DmWRNexo incubated with ss DNA and cations as indicated, with or without Mg^2+^. **c** DmWRNexo incubated with ss (SS, *upper panel*) or duplex (5′OV, *lower panel*) DNA substrate with 4 mM Mg^2+^ and increasing amounts of EDTA. In all cases, products were analyzed on 14% denaturing PAGE

Human WRN exonuclease requires two divalent metal ions to be coordinated by acidic residues within the active site (Perry et al. 2006); such residues are conserved in DmWRNexo (Fig. S3a). We verified that the standard buffer used (WRN exo buffer) was also optimal for DmWRNexo nuclease activity (Fig. S5), and it was therefore used to test the cation requirements for nuclease activity of WT and D229V DmWRNexo in the presence of magnesium, manganese, calcium or zinc, on both ss and 5′OV substrates (Fig. 4a, upper and lower panels, respectively). Both WT DmWRNexo and the D229V mutant were found to show a specific requirement for Mg^2+^ (Fig. 4a, lanes 2 and 3), with no activity seen for either protein with any of the other cations at 4 mM on either ss or duplex substrates (Fig. 4a, lanes 4–12 inclusive). Since WRNexo from *Arabidopsis* can utilise cation concentrations as low as 100 μM (Plchova et al. 2003), we tested the cleavage activity of DmWRNexo with 100 μM Mg^2+^ or Mn^2+^. Intriguingly, manganese at this lower concentration did support exonuclease activity of DmWRNexo (Fig. 4b, lanes 3 and 13), with a similar degree of cleavage detected as with the same low concentration of Mg^2+^ (Fig. 4b, lane 10). Partial inhibition of cleavage was detected when Mn^2+^ (100 μM or 4 mM) or Ca^2+^ (100 μM) were added in the presence of 4 mM Mg^2+^ (Fig. 4b, lanes 6, 8, 12, and 15) suggesting some form of cation competition for the enzyme active site or perhaps suboptimal cleavage in a ‘hetero-cation’ state. By contrast, zinc totally blocked enzyme activity, perhaps by outcompeting Mg^2+^ (Fig. 4b, compare lanes 2 and 7). Therefore, DmWRNexo cleaves DNA preferentially using Mg^2+^ even at low levels, with higher amounts permitting greater nuclease activity.

Further, there is an absolute requirement for cations for DmWRNexo, as no substrate cleavage was detected without metal ions present (Fig. 4b, lanes 1 and 9). Consistent with this, the stimulation of nuclease activity by 4 mM Mg^2+^ was abrogated by addition of the chelating agent EDTA in a concentration-dependent manner: as increasing amounts of EDTA sequestered the cation, there was loss of activity (Fig. 4c), with single-stranded DNA cleavage inhibited at 2 mM EDTA (Fig. 4c, upper panel) and duplex cleavage markedly blocked at 4 mM EDTA (Fig. 4c, lower panel). Note that at a 1:1 molar ratio of cation/chelator (Fig. 4c, lane 5), nearly all nuclease activity was lost.

### ATP inhibits DmWRNexo

**Fig. 5:**
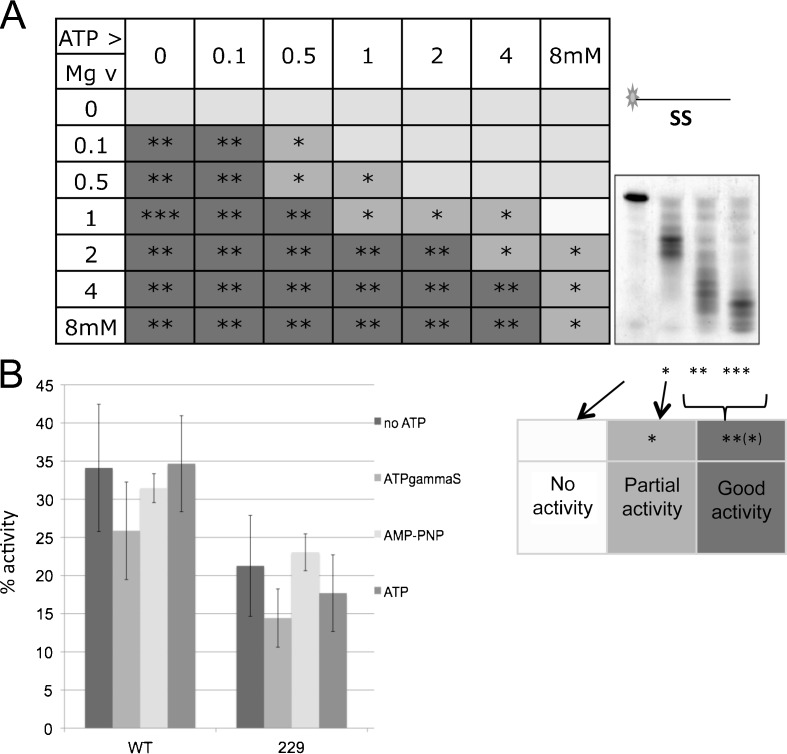
Inhibition of DmWRNexo nuclease activity by ATP. **a** Nuclease activity of 50 nM WT DmWRNexo on ss DNA with Mg^2+^ and/or ATP were scored as good, partial or none, as shown in the scheme on the right (*n* = 2). **b** 100 nM WT or D229V (229) DmWRNexo were tested on ss DNA substrate with 2 mM ATP, ATPγS, AMP-PNP or no ATP. Quantification of degradation assayed on denaturing gels used ImageJ as before (*n* = 3, ±SEM). (Note that removal of even a single nucleotide by D229V represents degradation of full-length substrate)

A physiological chelator of Mg^2+^ in cells is ATP, suggesting that ATP concentrations might impact on nuclease activity. Others have assessed human WRN exonuclease activity in the presence of 1 mM ATP, as this is required for the helicase activity intrinsic to hWRN (Shen et al. 1998). We therefore varied both ATP and Mg^2+^ concentrations from 100 μM to 8 mM; nuclease activity was assessed in terms of amount of product degraded and degree of degradation according to the schematic shown (Fig. 5a). As expected, in the absence of Mg^2+^, no cleavage was detected, while 100 μM Mg^2+^ supported good activity in the absence of ATP that was inhibited by increasing ATP concentrations. Greatest nuclease activity was observed at 1 mM Mg^2+^ in the absence of ATP (Fig. 5a). When ATP was added, more Mg^2+^ was required in the reaction, consistent with ATP sequestration of the cation; higher concentrations of ATP abrogated nuclease activity even at high cation concentrations, with an effective loss of activity at an ATP/Mg^2+^ molar ratio of between 1:1 and 2:1 for any concentration (Fig. 5a). This suggests that the ATP/Mg^2+^ ratio in an exonuclease buffer (and in the cell) is important for optimal activity. Note that the ATP/Mg^2+^ ratio in the WRN Exo/helicase buffer is 1:2 (Opresko et al. 2001) and so can effectively support both helicase and exonuclease activities of the human WRN protein.

### DmWRNexo does not require ATP hydrolysis to cleave DNA and lacks helicase activity

While our data strongly suggest that DmWRNexo does not require ATP (and has no helicase activity requiring it) but instead is inhibited by ATP (Fig. 5a and Fig. S6, lane 4), it has been reported elsewhere that hWRN exonuclease needs ATP to function (Machwe et al. 2006). To formally test the possibility that DmWRNexo requires ATP hydrolysis to act, we compared exonuclease function of both WT DmWRNexo and the attenuated D229V mutant on a single-stranded DNA substrate in the presence and absence of 2 mM ATP, AMP-PNP or ATPγS (non- or poorly-hydrolysable ATP analogues) with 4 mM Mg^2+^ (to avoid chelation of essential Mg^2+^). We did not detect any statistically significant differences in DmWRNexo nuclease activity between samples containing the various ATP analogues compared with controls lacking ATP (Fig. 5b).

### A novel active site mutant D222V lacks exonuclease activity

To further explore critical catalytic residues of DmWRNexo, we generated a novel mutation of aspartate 222 to valine, as this is predicted to lie also within the active site of the enzyme by comparison with hWRN exonuclease (see Fig. S3). The mutant D222V protein was expressed and purified to near-homogeneity (Fig. S7a, b) for functional testing *in vitro* in comparison with purified recombinant wild-type DmWRNexo. As predicted, DmWRNexo D222V lacked any exonuclease activity on either single-stranded DNA (Fig. S7c) or a 5′ overhang duplex substrate (data not shown), while further assessment of cleavage at different temperatures over an extended time course and in the presence of various cations detected no activity (Fig. S7c, d), suggesting that D222 is indeed a conserved co-ordinate catalytic residue.

To rule out the possibility that the desalting step was responsible for loss of enzyme activity of D222V, WT DmWRNexo, D222V or mock (negative control) imidazole eluates from His-Trap columns were tested directly in a standard nuclease reaction without desalting. Remarkably, wild-type DmWRNexo was exonucleolytically active even at 405 mM imidazole (Fig. S7e, f), suggesting it is a very robust enzyme with little sensitivity to salt; however, no activity was detected for D222V at any imidazole concentration tested. Hence, the mutant enzyme is likely to be inactive through loss of a critical aspartate in the active site, rather than through artefacts of enzyme preparation.

### DmWRNexo is active on alternative DNA structures including bubbles and recessed duplexes

Werner syndrome cells lacking functional WRN specifically show a defect in processing stalled replication forks (Rodriguez-Lopez et al. 2002; Sidorova et al. 2008). We therefore tested the ability of DmWRNexo to cleave substrates that might exist at sites of stalled or aborted DNA replication sites, or intermediates formed during processing of such sites. A range of mutants was assessed in comparison to wt DmWRNexo, including the novel D222V mutant, D229V surface mutation, and a double active site mutant ‘DE’ (Boubriak et al. 2009).

**Fig. 6:**
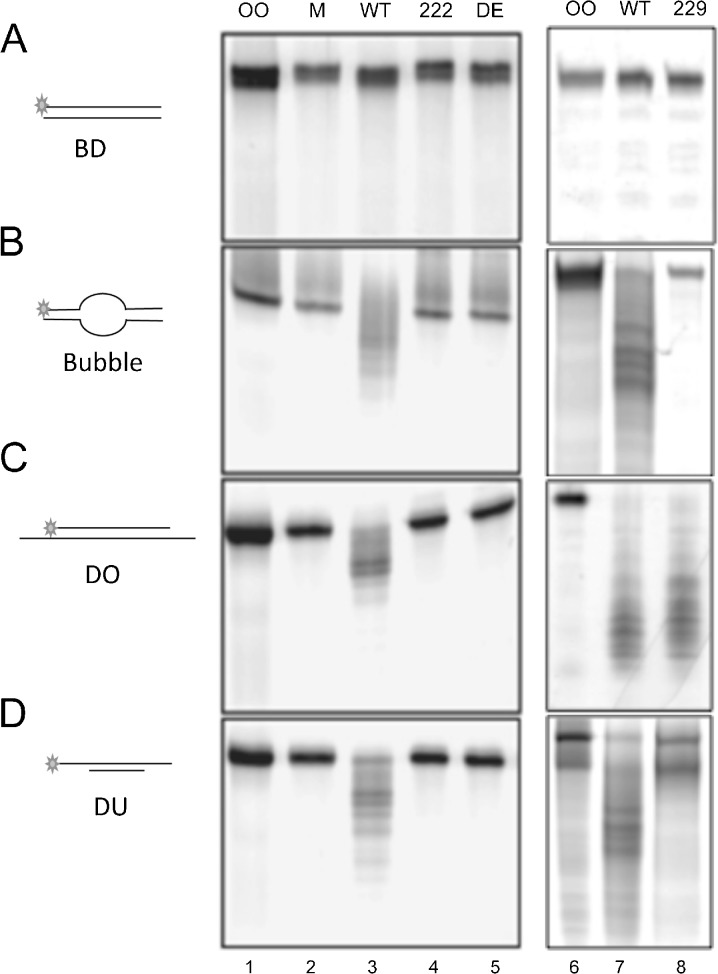
Cleavage of replication bubble and fork-like structures by DmWRNexo. Nuclease activity of 200 nM DmWRNexo [WT, the D222V (*222*), D162A E164A (*DE*) or D229V (*229*) mutants] or mock (*M*) negative control on 5′-labelled substrates: **a** blunt duplex (*BD*), **b** bubble substrate, **c** double overhang relative to the labelled strand (*DO*) and **d** double underhang relative to the labelled strand (*DU*). Products of nuclease activity were analysed on 14% denaturing PAGE after 30 min (*left panels*) or 45 min (*right panels*). (Note that SWT DmWRNexo is an internal comparator in all assays)

We found that DmWRNexo cannot cleave blunt ended duplex DNA (Fig. 6a). By contrast, extensive cleavage of a blunt-ended bubble substrate with an internal 20 nucleotide mismatched region was detected for the WT protein (Fig. 6b). These results together suggest that DmWRNexo requires ss DNA for loading, but it does not need a free single-stranded end.

Substrates with both a ‘double overhang’ (DO, Fig. 6c) and a ‘double underhang’ (DU, Fig. 6d) are equally well degraded by WT DmWRNexo, again supporting the assertion that single-stranded DNA is necessary for loading. Notably, the D229V mutant enzyme can cleave double overhang but not the double underhang substrates (compare lanes 8 in Fig. 6c and d), supporting the idea that the 5′-labelled strand acts to load the enzyme on the DU substrates and that the unlabelled strand is degraded first. Note that the D229V protein is probably blocked by fluorescein (see Fig. 3 and Fig. S2b), which may prevent its cleavage of the DU substrate. D229V also cannot degrade bubble substrates (Fig. 6b, lane 8) and may lack the structural flexibility necessary to bind onto constrained ss DNA.

### Activity of DmWRNexo on lesional templates

**Fig. 7:**
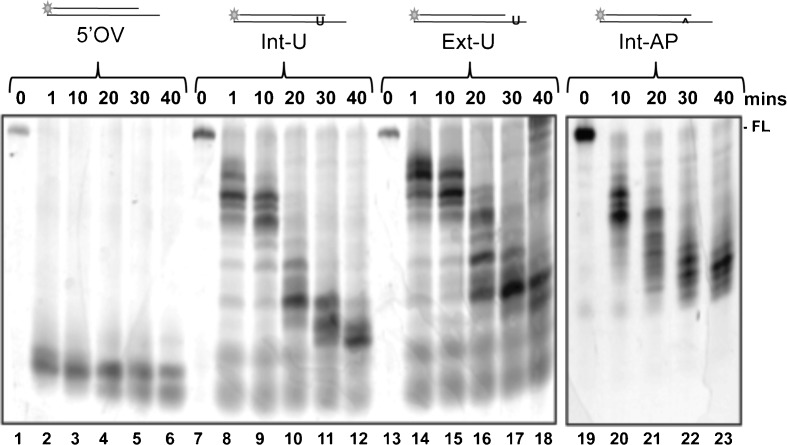
Inhibition of cleavage by uracil and abasic sites. The ability of DmWRNexo to degrade past a lesion was tested using 5′OV duplex substrate with a single uracil in the substrate tail (Ext-U) or an internal uracil or abasic site a short way into the duplex (Int-U or IntAP, respectively). Activity of WT DmWRNexo (200 nM) at 37°C for 0–40 min on 2 μM substrate is shown, with products analysed on 14% denaturing PAGE

WRN is implicated in base excision DNA repair (BER) (Bohr 2005; Harrigan et al. 2003, 2006), a process required to repair damage to individual bases, including removal of uracil from DNA. Thus, we tested cleavage of substrates containing a single uracil in the guide strand either external or internal to the duplex region (see Table 1 and Fig. 7). BER removes uracil using DNA glycosylase (UDG) resulting in an abasic site: we therefore mimicked this process by treating oligonucleotides containing uracil with UDG prior to annealing. Substrate integrity was verified on denaturing PAGE (Fig. S1b). Efficiency of conversion of uracil to AP sites by UDG was determined by treatment with potassium hydroxide to break DNA at AP sites, followed by analysis on ethidium bromide PAGE (Fig. S1c); virtually all the UDG-treated substrates were cleaved upon exposure to alkali. External AP sites proved extremely fragile and fragmented prior to use so were not employed.

As expected, WT DmWRNexo efficiently cleaved a 5′-labelled 5′ overhang substrate (5′OV, Fig. 7, lanes 1–6) compared to lesion-containing 5′OV substrates. Note that this cleavage is very efficient because high concentrations of enzyme (200 nM) were employed in these assays. While the internal uracil substrate was subject to some degradation, specific pause sites were detected such that even after 40-min incubation with high concentrations of enzyme, degradation did not proceed to completion (Fig. 7, lanes 8–12). This pausing was even more marked when the uracil was placed external to the duplex region in a position where presumably the enzyme binds initially to the single-stranded overhang. The position of pause sites correlated broadly with the position of the uracil. Interestingly, a single internal abasic site gave rise to the same initial pause sites as observed with internal uracil in the same position (compare Fig. 7, lanes 20 and 9), but a stop, rather than pause, site was subsequently encountered, resulting in DNA products of the same size as those observed with external uracil in the single-stranded region (compare Fig. 7, lanes 22 and 23 with lanes 17 and 18). Taken together, these data strongly suggest that DmWRNexo is incapable of degrading substrates that contain abasic sites and is severely inhibited by the presence of uracil, even though the lesion is in the guide strand and not in the degraded strand.

## Discussion

In this paper, we examine in detail the enzymology of the *Drosophila* orthologue of hWRN exonuclease, DmWRNexo. The ability of DmWRNexo to cleave single-stranded DNA with the high efficiency that we observe here is fully consistent with reports showing that hWRN exo degrades ssDNA in a length-dependent manner (Machwe et al. 2006; Xue et al. 2002). (Previous suggestions that hWRN is unable to cleave single-stranded DNA were based on substrates much shorter than the 50-mer oligonucleotide used here.) Given the probable roles of WRN exonuclease in end processing (Perry et al. 2006) during either classical DNA repair or in processing defective replication forks (e.g. Machwe et al. 2007; Rodriguez-Lopez et al. 2002, 2007; Sidorova et al. 2008), activity on ss DNA may be required. Notably, the RecQ family member human BLM, which is highly related to hWRN, can translocate along ssDNA (Gyimesi et al. 2010)—perhaps human WRN similarly translocates via its helicase activity but also can cleave through ss exonuclease activity.

Here, we clearly demonstrate that DmWRNexo, like hWRN exonuclease, is a 3′–5′ exonuclease. Furthermore, the enzyme is dependent upon Mg^2+^ (or low levels of Mn^2+^) for activity and has no requirement for ATP. Published hWRN crystal structures of the active site show identical spacing of 3.7 Å between the two metal ions, whether Mg^2+^ or Mn^2+^ (Perry et al. 2006); moreover, WRNexo from *Arabidopsis* can also utilize Mg^2+^ or Mn^2+^ (Plchova et al. 2003). DmWRNexo acts with 3′–5′ polarity since it progressively degrades 5′-labelled substrates to produce a ladder of products of decreasing size, while a 3′ label is immediately cleaved (see Fig. 3). These findings are incompatible with any 5′–3′ exonuclease activity. The 3′ fluorescein label might be anticipated to interfere with exonuclease cleavage since hWRN exonuclease does not cleave blocked 3′ ends bearing 3′ phosphate, 3′ phosphoglycolates or 3′ tyrosyl residues (Harrigan et al. 2007). However, DmWRNexo showed good activity on the 3′-labelled substrate, removing the 3′ FLO label from approximately 40% of the full-length substrate. Therefore, it is highly unlikely that fluorescein is specifically blocking a putative 5′–3′ activity, although it should be noted that while 40% of 3′-labelled substrate is degraded, around 80% of 5′-labelled substrate is cleaved under identical conditions, so the configuration of fluorescein presentation does impact to some extent on degradation. Taken together, our results here demonstrate that DmWRNexo is a *bona fide* 3′–5′ exonuclease.

The single nucleotide clipping by the D229V enzyme implies that once this mutant enzyme binds to substrate and cleaves off a single nucleotide, it remains bound to that substrate and cannot dissociate—if it could, then we would expect sequential binding, cleavage and dissociation resulting in progressive degradation of the substrate with time, though probably with slower kinetics than WT since the binding step rather than nucleotide hydrolysis is likely to be rate limiting. The mutation of a surface aspartate to valine in the D229V mutant may result in overall loss of protein stability, failure of oligomerisation or, more subtly, affect association of DNA with the enzyme, particularly if D229 acts as a guide residue to channel DNA to be degraded into the enzyme’s active site. Since D229 has some limited activity on duplex DNA, positioning of the non-degraded strand may allow processive repositioning of the enzyme whereas the lack of this guide strand on ssDNA substrates may cause blockage or non-reversible binding. The inability of this mutant protein to cleave substrates bearing a 3′ FLO is particularly interesting in this context; perhaps the D229V mutant lacks flexibility to accommodate the fluorescein group, or the path of DNA through the protein is altered.

Like hWRN exonuclease, DmWRNexo is inactive on duplex substrates with blunt ends, but is able to cleave bubble substrates. The human WRN enzyme requires ATP for activity on such substrates (Shen and Loeb 2000), though this is probably to support the helicase action in providing a suitable template for degradation by the nuclease activity. By contrast, we show that DmWRNexo does not require ATP for DNA degradation, but neither does it possess any ATP-dependent helicase domains, so a lack of ATP requirement is not unexpected. It is theoretically possible that cleavage of bubble substrates by DmWRNexo may involve a cryptic endonuclease activity, as has been suggested for hWRN (Xue et al. 2002). However, as we have seen no other evidence for endonuclease activity in this study, and DmWRNexo has activity on ssDNA, it is more likely that cleavage of bubbles by DmWRNexo results from the protein binding to ssDNA within the unpaired region of the bubble substrate. Intriguingly, the D229V variant is inactive on DNA bubbles or double underhang substrates. Such limited activity correlates with elevated levels of mitotic exchange in *CG7670* ^*e04496*^ */CG7670* ^*D229V*^ flies (Boubriak et al. 2009) and is fully consistent with an inability of the D229V mutant protein to bind to stalled fork substrates, which would result in the generation of double strand breaks, and their subsequent repair by cross-over pathways of homologous recombination.

hWRN exonuclease is inhibited by various oxidative lesions in either strand of a synthetic duplex (Bukowy et al. 2008; Harrigan et al. 2007) with around 50–70% decrease in activity, although hWRN *helicase* can unwind long-patch BER substrates (Harrigan et al. 2003) and is known to participate in BER downstream of the initial processing events (Harrigan et al. 2003, 2006). We show that DmWRNexo also degrades duplex substrates containing uracil in the guide strand, with a similar inhibition or ‘pausing’ at the lesions (while the pause site is strong, there is evidence of cleavage past the uracil site since smaller DNA products are detected below the major ‘pause’ band). A single abasic site instead inhibits any further substrate cleavage, possibly resulting in dissociation of the enzyme from its substrate. In humans, Ku stimulation overcomes this inhibition (Bukowy et al. 2008), suggesting that any role of DmWRNexo in BER may require co-operation with a WRN-like helicase and Ku orthologues, and is likely to be downstream of abasic-site processing.

The progeroid human Werner syndrome presents a useful model system to study the biology of ageing by investigating the role(s) of the protein WRN, the function of which is lost in WS. Despite such usefulness, human WS suffers from serious experimental limitations, particularly in the rarity and genetic heterogeneity of patient material, the inability to study the impact of chosen mutations on organismal phenotype and the confounding variable of the helicase co-existing on the same polypeptide as the nuclease (mutations may therefore have a dominant negative effect, e.g. Crabbe et al. 2004). To overcome these limitations, we are developing a Werner syndrome fly model. We have identified the *Drosophila* orthologue of human WRN exonuclease (Cox et al. 2007), hypomorphic mutation of which results in WS-like phenotypes including hypersensitivity to CPT and extremely high rates of recombination (Saunders et al. 2008), and shown that the protein does indeed possess exonuclease activity (Boubriak et al. 2009). Here, we have analysed the activity of this exonuclease, DmWRNexo. Our demonstration of 3′–5′ directionality, requirement for Mg^2+^, activity on replication-like substrates and inhibition by uracil and abasic sites show that DmWRNexo is enzymatically very similar to its human orthologue. We have additionally explored the impact both of active site mutations (essentially null) and of a more subtle alteration impacting on surface fold, that nevertheless has a marked negative effect on enzyme processivity and ability to cleave both ss DNA and replication-type substrates, particularly DNA bubbles. Such characteristics of DmWRNexo therefore provide strong validation of the fly model of WS and allow effects on the organism to be interpreted within the context of a clear biochemical understanding of the activity of the WRN nuclease.

## Electronic supplementary material

Below is the link to the electronic supplementary material.

Figure S1 Generation of DNA substrates. **a** Integrity of oligonucleotides for DNA substrates was verified by running 60 pmol of each substrate post-annealing on 10% native PAGE (1× TBE, 10% 19:1 acrylamide/bis-acrylamide) and visualised using a Fuji FLA-3000 analyser. **b** To make abasic (AP) sites, oligonucleotides containing a single uracil residue (Table 1) were treated with uracil DNA glycosylase (*Escherichia coli* UDG, Roche) at 10 U/nmol DNA for 16 h at 37°C. AP-containing duplex substrates were then prepared by annealing to the FLO strand and analysed as above. **c** The existence of AP sites was confirmed by conversion of the AP sites to breaks by incubating with 50 mM KOH at 60°C for 30 min, with separation on 12% native PAGE with ethidium bromide. *Ds* double stranded, *c* cut with uracil DNA glycosylase, *uc* uncut. (TIFF 150 kb) (JPEG 13 kb)

High Resolution Image 1 (TIFF 150 kb)

Figure S2 Quantification of nuclease activity. **a** Effect of increasing DmWRNexo protein concentration on degradation of ss and duplex product (*n* = 3, ±SEM). Logarithmic regressions are also shown (*dotted lines*; see text for *R* ^2^ values). **b** Degradation of 5′ or 3′ end-labelled duplex substrate by WT DmWRNexo (*n* = 3, ±SEM). (Nuclease activity was determined by separating products on denaturing PAGE, acquiring images using a Fuji FLA-3000 analyser and quantifying band intensity using ImageJ. Degradation is expressed as the percentage reduction in band intensity of full-length substrate, normalised to the oligonucleotide alone or zero time point.) See Figs. 1 and 3 for representative gels. (JPEG 19 kb)

High Resolution Image 2 (TIFF 192 kb)

Figure S3 DmWRNexo mutagenesis. **a** Alignment of catalytic core region of hWRN exonuclease domain and DmWRNexo showing D222 and the cognate D143 in hWRN, plus other residues selected for site-directed mutagenesis (*red boxes*): D162 and E164 (equivalent to hWRN exo D82 and E84), and D229 (equivalent to human D150). **b** Structural modelling of the predicted active site of DmWRNexo to show the impact of mutagenesis (*red arrows*); note that D229 lies outside this region and its mutation to valine has no predicted effect upon the configuration of residues at the catalytic core. **c** Predicted effect of mutation D229V on the surface of DmWRNexo compared with WT protein. Modelling was conducted using Swiss-Model and MacPymol. Surface mesh is shown in blue for WT and orange for D229V; the aspartate 229 (WT) and valine 229 (mutant) are shown in *pink*. (JPEG 38 kb)

High Resolution Image 3 (TIFF 2671 kb)

Figure S4 Comparison of nuclease polarities. Analysis of DmWRNexo against *E. coli* 3′–5′ Exo1 (Exo1) and 5′–3′ Lambda exonuclease (λ exo, NEB). 100 nM WT DmWRNexo or 10 U of commercially prepared exonucleases in 1× commercial Lambda exonuclease buffer (NEB) with 2 nM substrate for 30 min at 37°C. *Lanes 1–7*, ss substrate SS; *lanes 8–14*, duplex 5′ FLO 5′-tailed substrate 5′-OV. Note that lambda exonuclease is inactive on single-stranded substrates (*lane 7*). DmWRNexo activity was supported in both Exo and lambda buffers. DmWRNexo shows a ladder of degradation suggesting removal of nucleotides from the 3′ end of the labelled strand, as does Exo1 (showing greater activity). The pattern of degradation shown for lambda exo activity is consistent with the reverse polarity with the 5′ label being clipped off. (JPEG 19 kb)

High Resolution Image 4 (TIFF 167 kb)

Figure S5 Buffer dependencies of DmWRNexo. WT and D229V were tested for cleavage of 5′ FLO ss oligonucleotide substrate compared with commercial exonuclease (DNA ExoI from *E. coli*) in a range of buffers to determine the optimal buffer for DmWRNexo activity (ss DNA substrate). D229V was also tested on a duplex 5′OV substrate. ‘Exo’ buffer: WRN exonuclease buffer (40 mM Tris–HCl, pH 8.0, 4 mM MgCl_2_, 5 mM dithiothreitol, 0.1 mg/ml BSA; Opresko et al. 2001); ‘RecQ’ buffer (optimised for RecQ helicase activity): 66 mM sodium acetate, 33 mM Tris–acetate pH 7.8, 100 μg/ml BSA, 1 mM DTT (Bachrati and Hickson 2006) and ‘DmExo’ buffer (optimised for fly exonuclease activity—50 mM Tris–Cl pH 8.0, 10 mM NaCl, 5 mM MgCl_2_, 0.2 mM EDTA and 50 μg/ml BSA) (Sander and Benhaim 1996), commercial ‘Exo1’ buffer (NEB). WRN exo buffer was shown to support activity significantly greater than the other three buffers tested (*n* = 3 ±SEM, *asterisk* indicates *P ≤* 0.05, Student’s *T* test). (JPEG 9 kb)

High Resolution Image 5 (TIFF 66 kb)

Figure S6 Testing helicase activity of DmWRNexo proteins. DmWRNexo (WT/mutants) WRN ‘Exo’ buffer ±2 mM ATP in a final volume of 20 μl. **a** Helicase activity: 5 μl helicase stop buffer (16.67 mM EDTA, 13.33% glycerol, 0.3% SDS final) was added to half of the reaction mix and immediately cooled to 4°C. No helicase activity was detected for WT DmWRNexo or the D229V or DE mutants (*M* = mock negative control; 8% SDS–PAGE; 1× TBE, 8% 19:1 acrylamide/bis-acrylamide, 0.1% SDS, 150 V 150 min). **b** Nuclease activity: at the end of incubation, the remaining half of each reaction mix was treated with formamide stop dye (14% denaturing PAGE). 2 mM ATP inhibited exonuclease activity such that no degradation products were seen for WT DmWRNexo (compare *lanes 3* and *4* for each gel). The high mobility products detected in *lane 3* with DmWRNexo reflect exonuclease degradation of the substrate rather than any possible helicase activity since the reaction was inhibited (not stimulated) by ATP (*lane 4*), and was only detected with WT DmWRNexo and not with any of the proteins mutated in nuclease active site residues. Thus, we can rule out the possibility of DmWRNexo having cryptic helicase activity, and moreover, these results also demonstrate that there is no low abundance, high activity helicase from *E. coli* co-purifying with DmWRNexo. (JPEG 13 kb)

High Resolution Image 6 (TIFF 159 kb)

Figure S7 Purification and exonuclease assay of D222V mutant. **a** Hexa-His tagged D222V expressed from pIVEX2.3d in *E. coli* BL21 T7 I^q^ LysY (NEB) purified on 1 ml HisTrap column (Amersham) with imidazole elution, as described previously (Boubriak et al. 2009). *M* marker, *L* load (input), *FT* flow-through, *W* wash, *numbered lanes* denote fraction number at the indicated concentration of imidazole, *plus sign* (+) indicates positive control (purified WT DmWRNexo). **b** Desalted purified proteins (Coomassie-stained 12% SDS–PAGE; 50 pmol WT, 100 pmol D222V). **c** Time course of D222V nuclease activity on ss DNA substrates (see Boubriak et al. 2009 for comparison with WT). **d** D222V nuclease activity with divalent cations (all at 4 mM) as indicated. **e** Exonuclease activity on 5′OV duplex substrate of His-Trap column fractions for WT, D222V and mock negative control (vector only) versus Exo1 positive control (NEB), without desalting; final imidazole concentrations are shown. **f** Quantification of exonuclease activity as in (**e**) (*n* = 2, ±SEM). Note that WT DmWRNexo is active even at high concentrations of imidazole. (JPEG 45 kb)

High Resolution Image 7 (TIFF 505 kb)
