# Supplementary figures and images for: The Drosophila orthologue of progeroid human WRN exonuclease, DmWRNexo, cleaves replication substrates but is inhibited by uracil or abasic sites: Analysis of DmWRNexo activity in vitro
Source: Age (Dordr). 2012 May 5;35(3):793–806. doi: 10.1007/s11357-012-9411-0 (PMC3636389; doi:10.1007/s11357-012-9411-0)

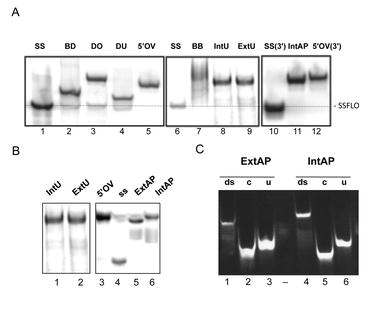

Supplement: Supplementary file 1 — Generation of DNA substrates. a Integrity of oligonucleotides for DNA substrates was verified by running 60 pmol of each substrate post-annealing on 10% native PAGE (1× TBE, 10% 19:1 acrylamide/bis-acrylamide) and visualised using a Fuji FLA-3000 analyser. b To make abasic (AP) sites, oligonucleotides containing a single uracil residue (Table 1) were treated with uracil DNA glycosylase (Escherichia coli UDG, Roche) at 10 U/nmol DNA for 16 h at 37°C. AP-containing duplex substrates were then prepared by annealing to the FLO strand and analysed as above. c The existence of AP sites was confirmed by conversion of the AP sites to breaks by incubating with 50 mM KOH at 60°C for 30 min, with separation on 12% native PAGE with ethidium bromide. Ds double stranded, c cut with uracil DNA glycosylase, uc uncut. (TIFF 150 kb) (JPEG 13 kb) [file 11357_2012_9411_Fig8_ESM.jpg]

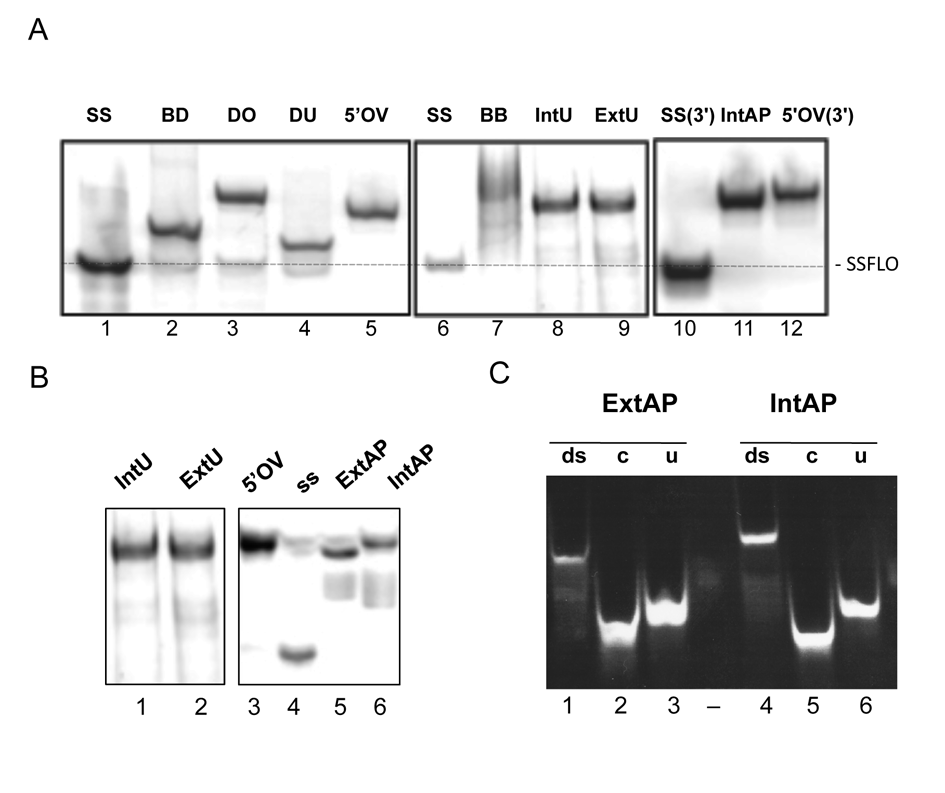

Supplement: Supplementary file 2 — (TIFF 150 kb) [file 11357_2012_9411_MOESM1_ESM.tif]

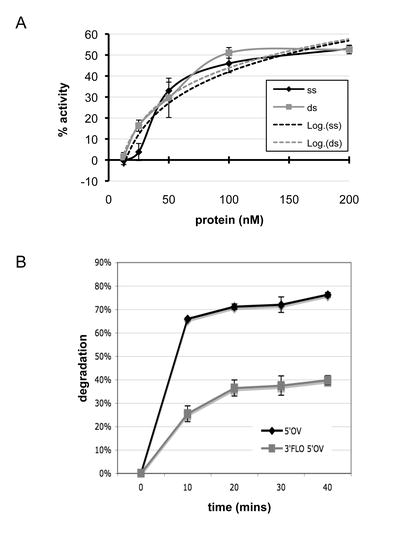

Supplement: Supplementary file 3 — Quantification of nuclease activity. a Effect of increasing DmWRNexo protein concentration on degradation of ss and duplex product (n = 3, ±SEM). Logarithmic regressions are also shown (dotted lines; see text for R 2 values). b Degradation of 5′ or 3′ end-labelled duplex substrate by WT DmWRNexo (n = 3, ±SEM). (Nuclease activity was determined by separating products on denaturing PAGE, acquiring images using a Fuji FLA-3000 analyser and quantifying band intensity using ImageJ. Degradation is expressed as the percentage reduction in band intensity of full-length substrate, normalised to the oligonucleotide alone or zero time point.) See Figs. 1 and 3 for representative gels. (JPEG 19 kb) [file 11357_2012_9411_Fig9_ESM.jpg]

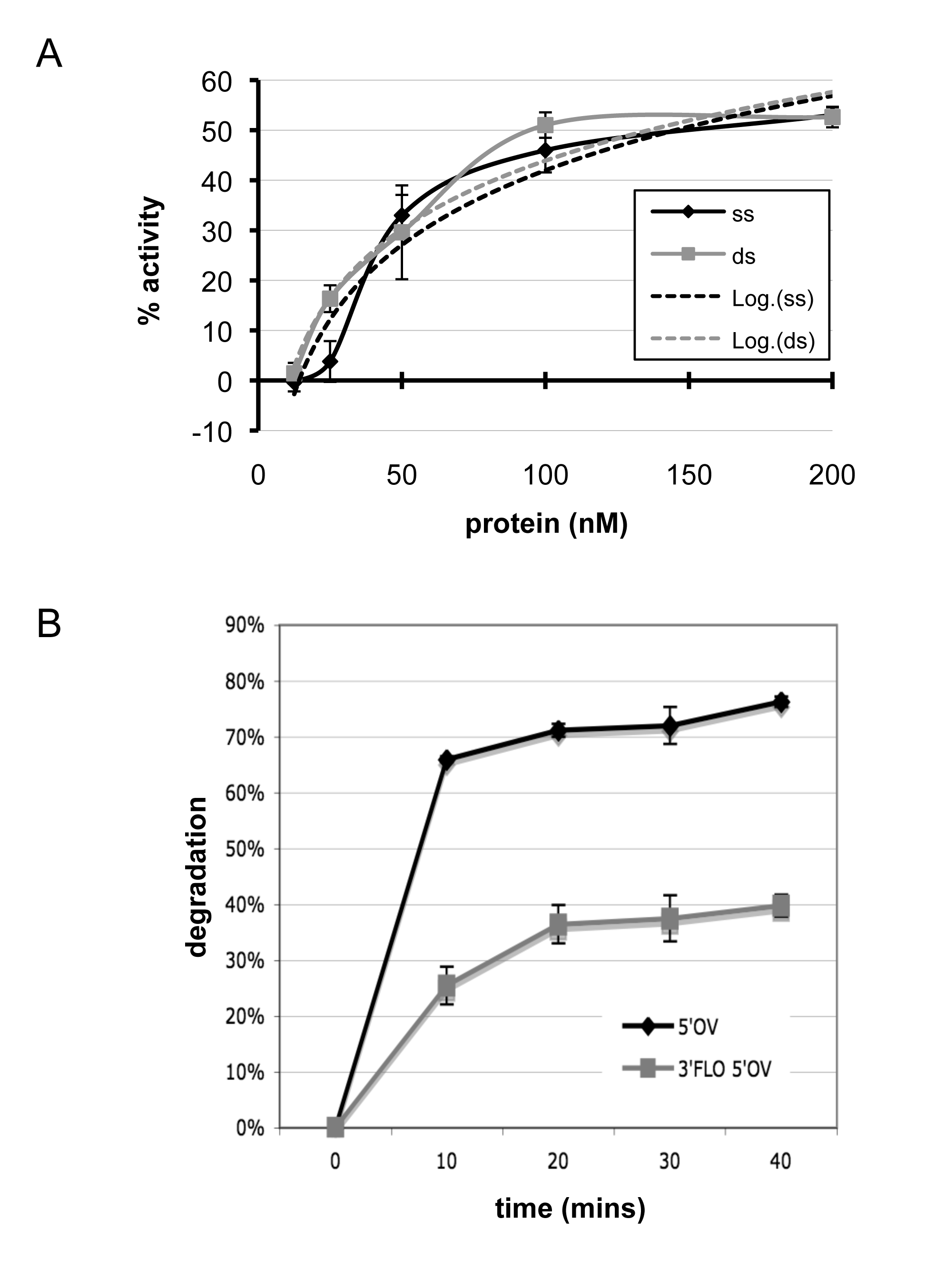

Supplement: Supplementary file 4 — (TIFF 192 kb) [file 11357_2012_9411_MOESM2_ESM.tif]

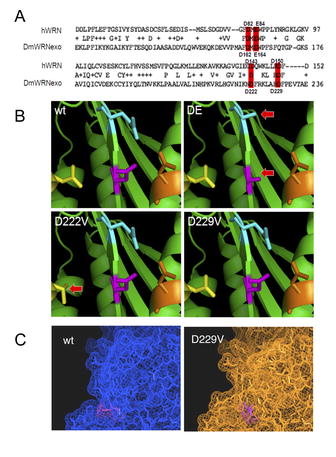

Supplement: Supplementary file 5 — DmWRNexo mutagenesis. a Alignment of catalytic core region of hWRN exonuclease domain and DmWRNexo showing D222 and the cognate D143 in hWRN, plus other residues selected for site-directed mutagenesis (red boxes): D162 and E164 (equivalent to hWRN exo D82 and E84), and D229 (equivalent to human D150). b Structural modelling of the predicted active site of DmWRNexo to show the impact of mutagenesis (red arrows); note that D229 lies outside this region and its mutation to valine has no predicted effect upon the configuration of residues at the catalytic core. c Predicted effect of mutation D229V on the surface of DmWRNexo compared with WT protein. Modelling was conducted using Swiss-Model and MacPymol. Surface mesh is shown in blue for WT and orange for D229V; the aspartate 229 (WT) and valine 229 (mutant) are shown in pink. (JPEG 38 kb) [file 11357_2012_9411_Fig10_ESM.jpg]

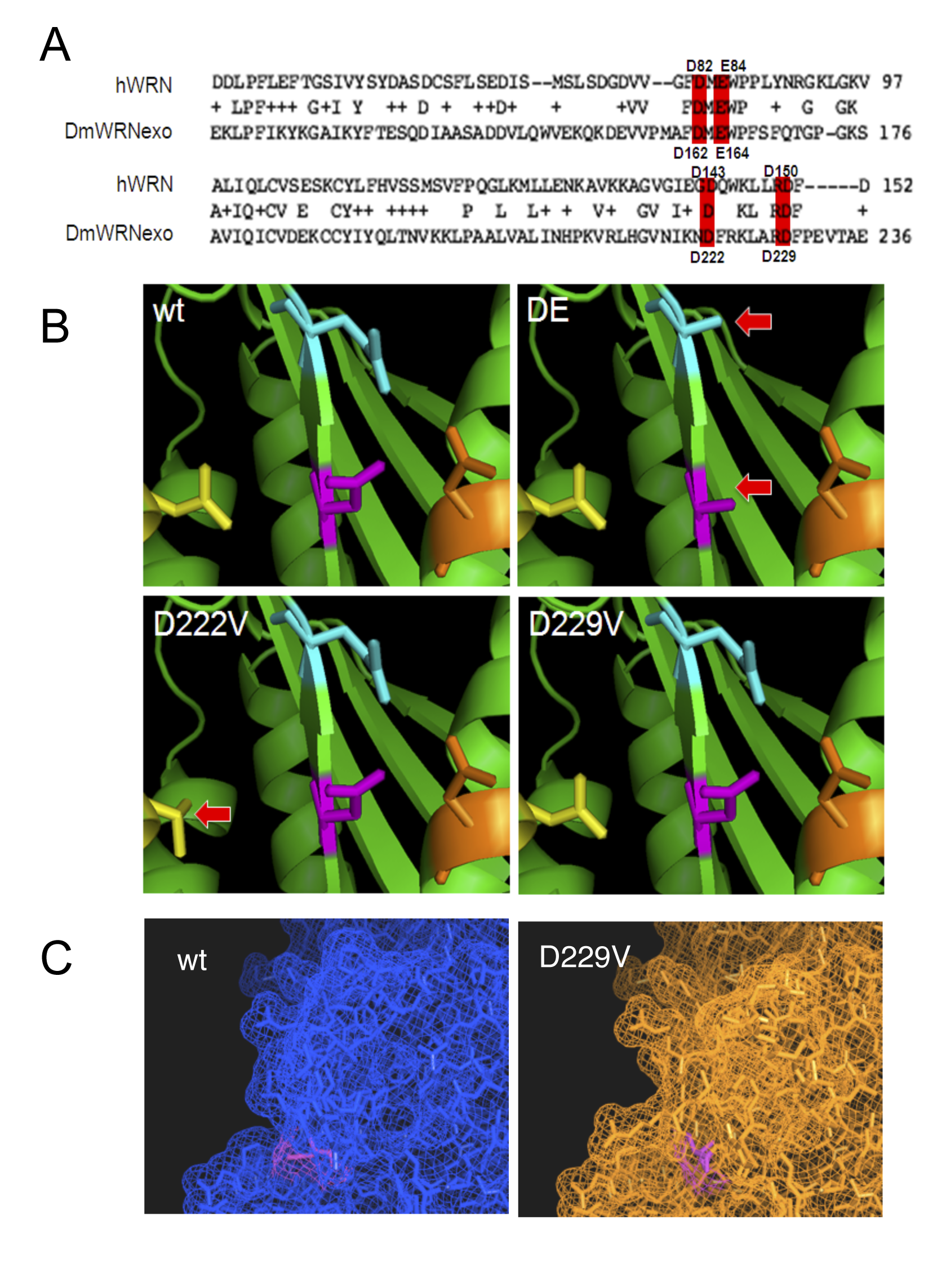

Supplement: Supplementary file 6 — (TIFF 2671 kb) [file 11357_2012_9411_MOESM3_ESM.tif]

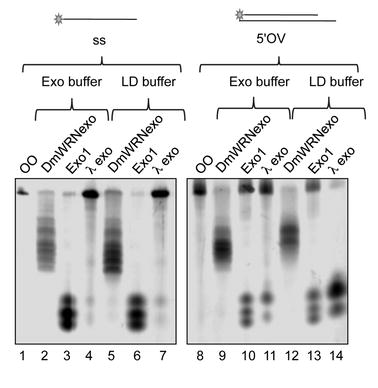

Supplement: Supplementary file 7 — Comparison of nuclease polarities. Analysis of DmWRNexo against E. coli 3′–5′ Exo1 (Exo1) and 5′–3′ Lambda exonuclease (λ exo, NEB). 100 nM WT DmWRNexo or 10 U of commercially prepared exonucleases in 1× commercial Lambda exonuclease buffer (NEB) with 2 nM substrate for 30 min at 37°C. Lanes 1–7, ss substrate SS; lanes 8–14, duplex 5′ FLO 5′-tailed substrate 5′-OV. Note that lambda exonuclease is inactive on single-stranded substrates (lane 7). DmWRNexo activity was supported in both Exo and lambda buffers. DmWRNexo shows a ladder of degradation suggesting removal of nucleotides from the 3′ end of the labelled strand, as does Exo1 (showing greater activity). The pattern of degradation shown for lambda exo activity is consistent with the reverse polarity with the 5′ label being clipped off. (JPEG 19 kb) [file 11357_2012_9411_Fig11_ESM.jpg]

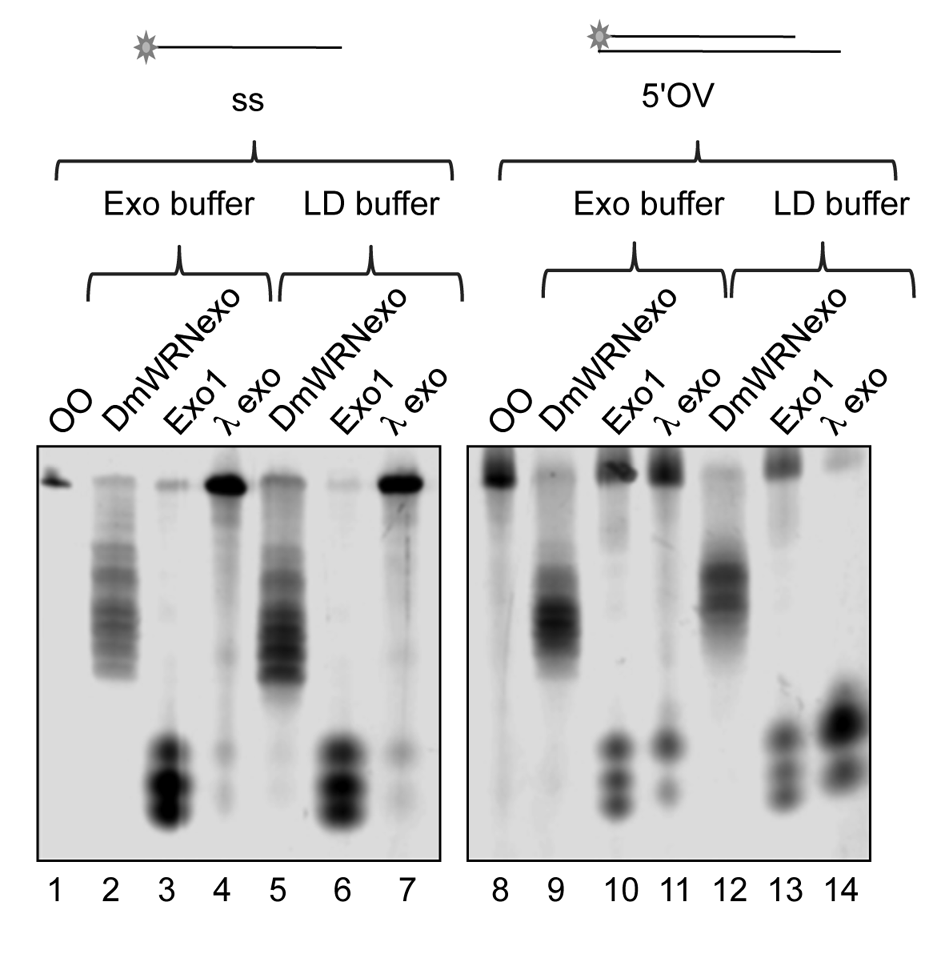

Supplement: Supplementary file 8 — (TIFF 167 kb) [file 11357_2012_9411_MOESM4_ESM.tif]

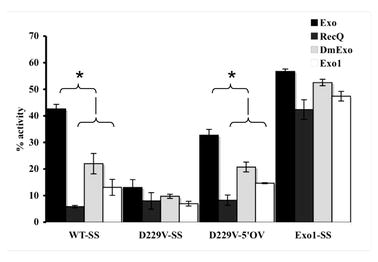

Supplement: Supplementary file 9 — Buffer dependencies of DmWRNexo. WT and D229V were tested for cleavage of 5′ FLO ss oligonucleotide substrate compared with commercial exonuclease (DNA ExoI from E. coli) in a range of buffers to determine the optimal buffer for DmWRNexo activity (ss DNA substrate). D229V was also tested on a duplex 5′OV substrate. ‘Exo’ buffer: WRN exonuclease buffer (40 mM Tris–HCl, pH 8.0, 4 mM MgCl2, 5 mM dithiothreitol, 0.1 mg/ml BSA; Opresko et al. 2001); ‘RecQ’ buffer (optimised for RecQ helicase activity): 66 mM sodium acetate, 33 mM Tris–acetate pH 7.8, 100 μg/ml BSA, 1 mM DTT (Bachrati and Hickson 2006) and ‘DmExo’ buffer (optimised for fly exonuclease activity—50 mM Tris–Cl pH 8.0, 10 mM NaCl, 5 mM MgCl2, 0.2 mM EDTA and 50 μg/ml BSA) (Sander and Benhaim 1996), commercial ‘Exo1’ buffer (NEB). WRN exo buffer was shown to support activity significantly greater than the other three buffers tested (n = 3 ±SEM, asterisk indicates P ≤ 0.05, Student’s T test). (JPEG 9 kb) [file 11357_2012_9411_Fig12_ESM.jpg]

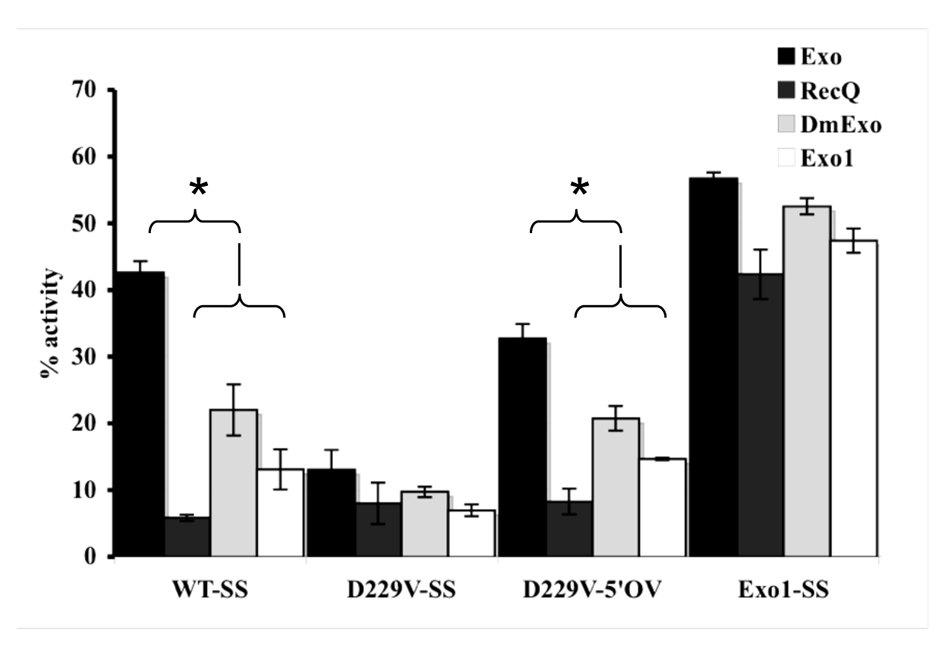

Supplement: Supplementary file 10 — (TIFF 66 kb) [file 11357_2012_9411_MOESM5_ESM.tif]

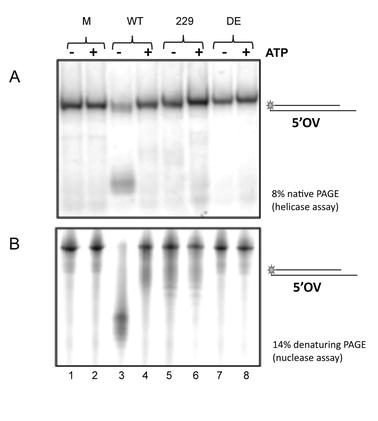

Supplement: Supplementary file 11 — Testing helicase activity of DmWRNexo proteins. DmWRNexo (WT/mutants) WRN ‘Exo’ buffer ±2 mM ATP in a final volume of 20 μl. a Helicase activity: 5 μl helicase stop buffer (16.67 mM EDTA, 13.33% glycerol, 0.3% SDS final) was added to half of the reaction mix and immediately cooled to 4°C. No helicase activity was detected for WT DmWRNexo or the D229V or DE mutants (M = mock negative control; 8% SDS–PAGE; 1× TBE, 8% 19:1 acrylamide/bis-acrylamide, 0.1% SDS, 150 V 150 min). b Nuclease activity: at the end of incubation, the remaining half of each reaction mix was treated with formamide stop dye (14% denaturing PAGE). 2 mM ATP inhibited exonuclease activity such that no degradation products were seen for WT DmWRNexo (compare lanes 3 and 4 for each gel). The high mobility products detected in lane 3 with DmWRNexo reflect exonuclease degradation of the substrate rather than any possible helicase activity since the reaction was inhibited (not stimulated) by ATP (lane 4), and was only detected with WT DmWRNexo and not with any of the proteins mutated in nuclease active site residues. Thus, we can rule out the possibility of DmWRNexo having cryptic helicase activity, and moreover, these results also demonstrate that there is no low abundance, high activity helicase from E. coli co-purifying with DmWRNexo. (JPEG 13 kb) [file 11357_2012_9411_Fig13_ESM.jpg]

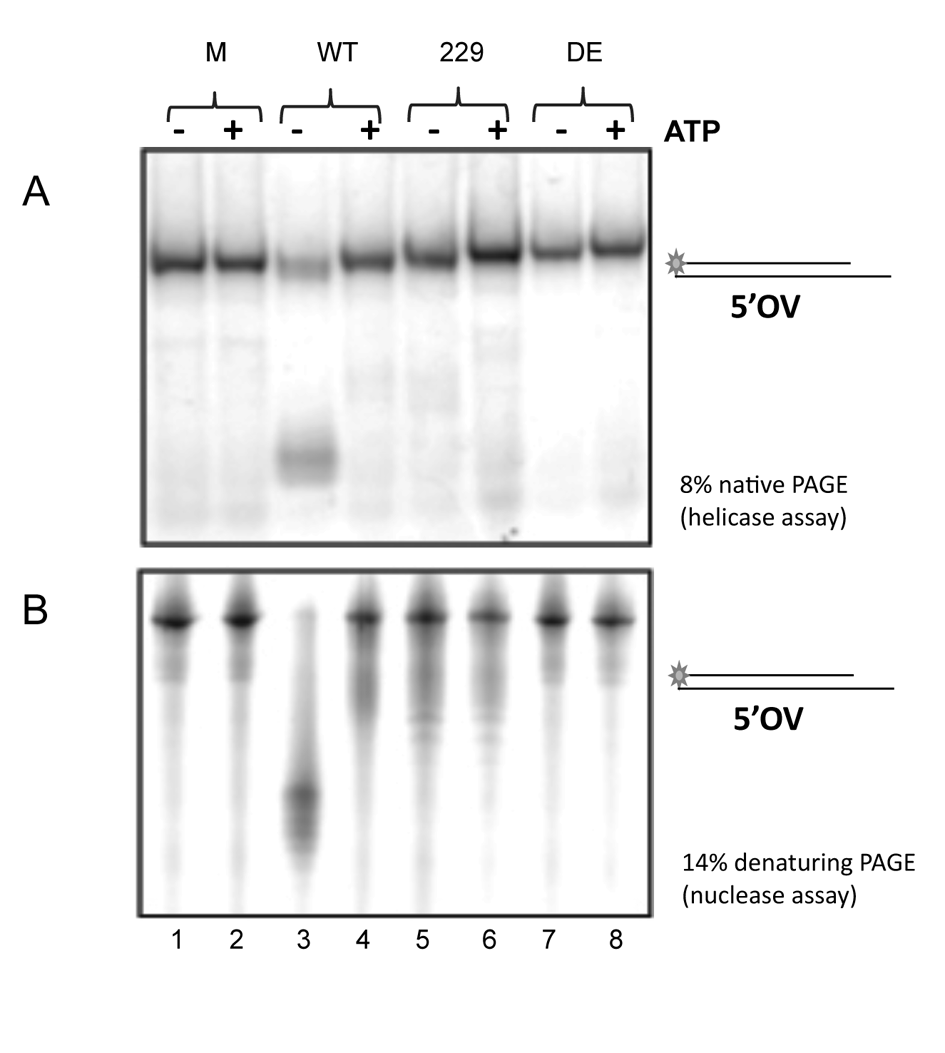

Supplement: Supplementary file 12 — (TIFF 159 kb) [file 11357_2012_9411_MOESM6_ESM.tif]

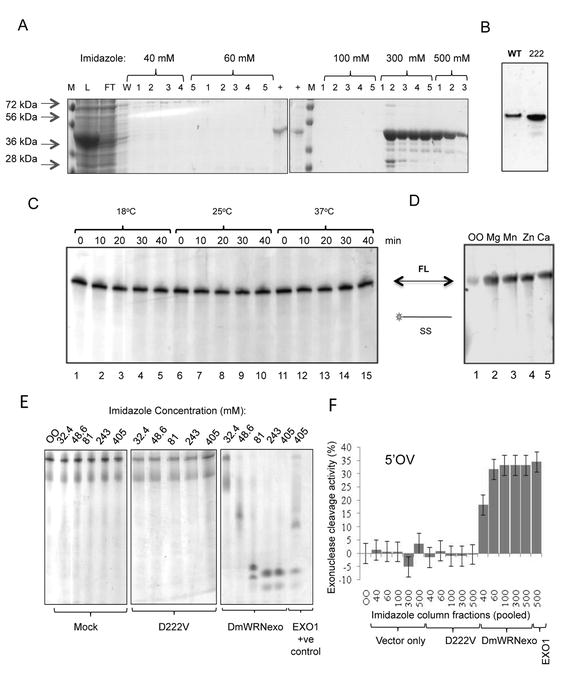

Supplement: Supplementary file 13 — Purification and exonuclease assay of D222V mutant. a Hexa-His tagged D222V expressed from pIVEX2.3d in E. coli BL21 T7 Iq LysY (NEB) purified on 1 ml HisTrap column (Amersham) with imidazole elution, as described previously (Boubriak et al. 2009). M marker, L load (input), FT flow-through, W wash, numbered lanes denote fraction number at the indicated concentration of imidazole, plus sign (+) indicates positive control (purified WT DmWRNexo). b Desalted purified proteins (Coomassie-stained 12% SDS–PAGE; 50 pmol WT, 100 pmol D222V). c Time course of D222V nuclease activity on ss DNA substrates (see Boubriak et al. 2009 for comparison with WT). d D222V nuclease activity with divalent cations (all at 4 mM) as indicated. e Exonuclease activity on 5′OV duplex substrate of His-Trap column fractions for WT, D222V and mock negative control (vector only) versus Exo1 positive control (NEB), without desalting; final imidazole concentrations are shown. f Quantification of exonuclease activity as in (e) (n = 2, ±SEM). Note that WT DmWRNexo is active even at high concentrations of imidazole. (JPEG 45 kb) [file 11357_2012_9411_Fig14_ESM.jpg]

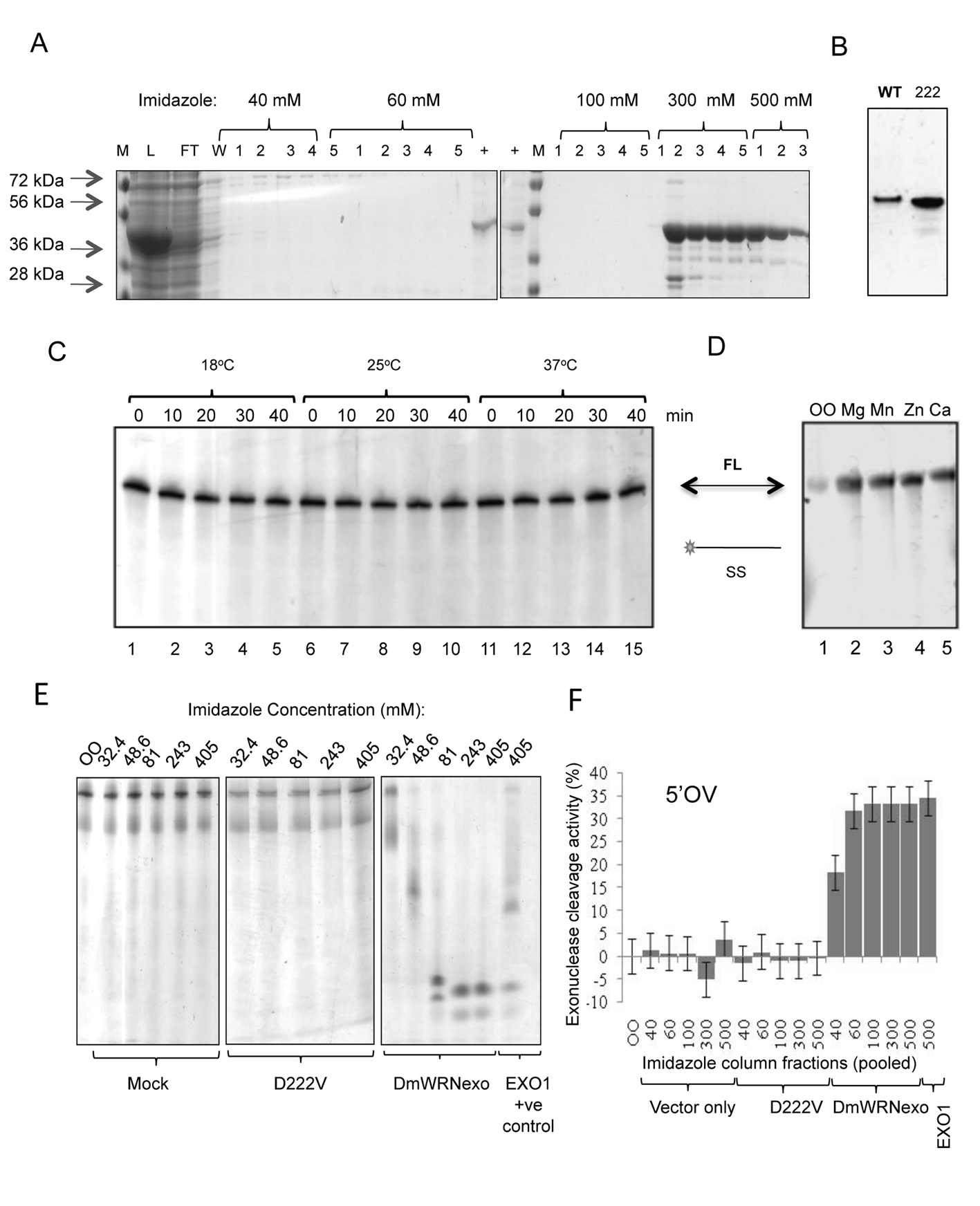

Supplement: Supplementary file 14 — (TIFF 505 kb) [file 11357_2012_9411_MOESM7_ESM.tif]
